# Complete Genome Sequence of Paludibaculum fermentans P105^T^, a Facultatively Anaerobic Acidobacterium Capable of Dissimilatory Fe(III) Reduction

**DOI:** 10.1128/MRA.01313-20

**Published:** 2021-01-07

**Authors:** Svetlana N. Dedysh, Alexey V. Beletsky, Irina S. Kulichevskaya, Andrey V. Mardanov, Nikolai V. Ravin

**Affiliations:** a Winogradsky Institute of Microbiology, Research Center of Biotechnology of the Russian Academy of Sciences, Moscow, Russia; b Institute of Bioengineering, Research Center of Biotechnology of the Russian Academy of Sciences, Moscow, Russia; University of Southern California

## Abstract

Paludibaculum fermentans P105^T^ is a facultatively anaerobic heterotroph of the phylum *Acidobacteria* which is capable of dissimilatory Fe(III) reduction. This bacterium is a common inhabitant of wetlands and groundwater bodies. The finished genome of strain P105^T^ is 9.53 Mb in size and contains multiple genes coding for membrane-bound multiheme cytochromes.

## ANNOUNCEMENT

Paludibaculum fermentans P105^T^ is a facultatively anaerobic bacterium of the order *Bryobacterales* (former subdivision 3 *Acidobacteria*), which, at present, comprises only three described representatives (1). It was isolated from the littoral wetland of a boreal lake located on Valaam Island, Karelia, northern Russia (2). Cells of strain P105^T^ are Gram-negative, nonmotile rods that occur singly or in short chains (2). These bacteria are mildly acidophilic and mesophilic heterotrophs, which inhabit wetlands and groundwater bodies (3). The key feature that differentiates Paludibaculum fermentans P105^T^ from other described members of the *Bryobacterales* is its ability to couple Fe(III) reduction to the fermentation of sugars and polysaccharides under anoxic conditions (2).

Strain P105^T^ was cultivated in a liquid mineral medium with glucose, as described earlier (2). DNA was extracted from the cells using the SDS-cetyltrimethylammonium bromide (CTAB) method (4) and used for genome sequencing by means of a combined application of pyrosequencing and Nanopore sequencing techniques. Genomic DNA was sequenced with a Roche genome sequencer FLX (GS FLX), using the TitaniumXL+ protocol for a shotgun library, resulting in the generation of 638,496 reads with an average read length of 530 bp. Quality trimming of the reads was done during base calling with 454 data processing software v3.0. The reads were *de novo* assembled into 126 contigs longer than 500 bp using the Newbler assembler v3.0 (454 Life Sciences, Branford, CT). For Nanopore sequencing, the library was prepared using the one-dimensional (1D) ligation sequencing kit (SQK-LSK108; Oxford Nanopore, UK). Sequencing of this library in an R9.4 flow cell (FLO-MIN106) using a MinION device yielded 152,011 reads with a total length of 664 Mb. The raw read *N*_50_ value was 23,671 bp, the average read length was 4,367 bp, and the maximum read length was 126,455 bp. Quality filtering was done during base calling using Albacore v2.3.1. Nanopore reads were corrected and trimmed using Canu v1.6 (5). The GS FLX contigs were merged into two circular sequences; gaps between the contigs were filled with trimmed and corrected Nanopore reads using npScarf v1.0 (6). To correct possible errors, the GS FLX reads were mapped to the assembly using Bowtie 2 v2.3.5.1 (7), and the consensus sequence was corrected using Pilon v1.22 (8). Default parameters were used for all software. The final assembly included a 9,476,802-bp chromosome and one circular plasmid of 55,292 bp. The assembled chromosome and plasmid sequences were annotated using the NCBI Prokaryotic Genome Annotation Pipeline. In total, 7,469 protein-coding genes were predicted in the genome.

Analysis of metabolic pathways indicated that *P. fermentans* P105^T^ is a facultatively anaerobic heterotroph capable of using complex polysaccharides, including cellulose, as growth substrates. The ability for dissimilatory Fe(III) reduction under anaerobic conditions is consistent with the presence of multiple genes coding for membrane-bound multiheme cytochromes.

### Data availability.

This whole-genome project has been deposited in GenBank under the accession number PRJNA672985. The GS FLX and Nanopore reads are available in the SRA under accession numbers SRX9395395 and SRX9395396, respectively. The genome sequence has been deposited under the accession numbers CP063849 (chromosome) and CP063850 (plasmid).
